# Analysis of Interleukin-17, Interleukin-23, neopterin and Nesfatin-1 levels in the sera of Hashimoto patients

**DOI:** 10.5937/jomb0-40683

**Published:** 2023-08-25

**Authors:** Nihayet Bayraktar, Mehmet Ali Eren, Mustafa Bayraktar, Ali Öztürk, Hamza Erdoğdu

**Affiliations:** 1 Harran University, Faculty of Medicine, Department of Medical Biochemistry, Şanlıurfa, Turkey; 2 Harran University, Faculty of Medicine, Department of Endocrinology, Şanlıurfa, Turkey; 3 Yıldırım Beyazıt University, Faculty of Medicine, Department of Internal Medicine, Ankara, Turkey; 4 Niğde Ömer Halisdemir University, Faculty of Medicine, Department of Medical Microbiology, Niğde, Turkey; 5 Harran University, Faculty of Business Administration, Department of Statistics, Şanlıurfa, Turkey

**Keywords:** Hashimoto's thyroiditis, Interleukin-17, Interleukin-23, Nesfatin-1, Neopetrin, Hashimoto tireoiditis, interlekin-17, interleukin-23, nesfatin-1, neopterin

## Abstract

**Background:**

Hashimoto's thyroiditis (HT) is an autoimmune disorder affecting the thyroid gland and may present as goiter or atrophic thyroiditis that may result in various metabolic and inflammatory disorders. The aim of this study is to determine the changes in serum levels of interleukin-17 (IL-17), IL-23, neopterin, and nesfatin-1 parameters in HT patients and to evaluate the possible relationship among these parameters.

**Methods:**

90 HT patients and 30 healthy individuals were included in this study. Demographic data of the patients included in the study were recorded and detailed physical examinations were performed. IL-17, IL-23, neopterin, and nesfatin-1 levels were measured in the serum samples of the participants by the ELISA method.

## Abstract (sr)

**Uvod:**

Hašimotov tiroiditis (HT) je autoimuni poremećaj koji utiče na štitnu žlezdu i može se manifestovati kao struma ili atrofični tiroiditis koji može dovesti do različitih metaboličkih i inflamatornih poremećaja. Cilj ovog istraživanja je da se utvrde promene u serumskim vrednostima parametara interleukina-17 (IL-17), IL-23, neopterina i nesfatina-1 kod pacijenata sa HT i da se proceni moguća povezanost ovih parametara.

**Metode:**

U ovu studiju je uključeno 90 pacijenata sa HT i 30 zdravih osoba. Snimani su demografski podaci pacijenata uključenih u studiju i obavljeni su detaljni fizički pregledi. Nivoi IL-17, IL-23, neopterina i nesfatina-1 mereni su u uzorcima seruma učesnika ELISA metodom.

**Rezultati:**

Serumski IL-17, IL-23, neopterin, nivoi su bili značajno viši i nivoi nesfatina-1 su bili značajno niži u HT grupi u poređenju sa kontrolnom grupom. Uočene su statistički značajne razlike između svih grupa osim eutireoidnog HT u odnosu na HT lečenih levotiroksinom za nesfatin-1 na nivou od 0,05.

**Zaključak:**

IL-17, IL-23 i neopterin su bili uključeni u etiopatogenezu HT. Povećanje telesne težine kod HT može biti posledica niskog nivoa nesfatina-1, a ne nedostatka hormona štitne žlezde. Ove varijable se mogu koristiti kao potencijalni biomarkeri u dijagnozi, prognozi i praćenju HT bolesti.

## Introduction

Hashimoto’s thyroiditis (HT) is one of the most common and organ-specific autoimmune diseases and it’s caused by the loss of immune tolerance of the thyroid gland [1]. It is characterized by the presence of thyroid autoantibodies such as thyroid peroxidase antibodies (TPO-Ab) and thyroglobulin antibodies (TG-Ab), which cause destruction of thyroid tissues leading to varying degrees of thyroid dysfunction [2] [3]. The actual pathogenesis is still unclear although genetic, hormonal and environmental factors are involved in the pathology of HT [4]. The most accepted theory of HT disease progression is the impaired immune response theory HT. The dysfunction of Suppressor-T lymphocytes leads to the production of thyroid tissue-specific autoantibodies from B cells stimulated by T-helper lymphocytes, and ultimately causes damage to thyroid cells [4] [5]. Untreated HT patients can lead to the development of papillary thyroid cancer and thyroid carcinoma [2] [6]. HT is also one of the factors leading to the development of hypertension, cardiovascular diseases, dyslipidemia, obesity, insulin resistance and depression [7] [8] [9].

Identification of a new subtype of T helper cells producing the IL-17 modified model of the Th1-Th2 paradigm and termed Th17 cells. The latter have high ability to stimulate acute and chronic inflammation making them an important role in the development of autoimmune disorders [10]. Numerous publications based on animal and human models confirm their important roles in the pathogenesis of human systemic and organ-specific autoimmune diseases [11].

IL-23 is an inflammatory cytokine and has been found to be essential for disease development in various autoimmune disease models [12]. It has been reported that increased serum levels of IL-23 in HT patients play a role in the development of HT by stimulating Th17 cell differentiation and IL-17 secretion [13]. A study has found that thyroid follicular cells from HT patients secrete high levels of IL-23, which contributes to autophagy suppression and neopterin accumulation [14].

Neopterin is produced by macrophages and responsible for activation the cellular immune response [15] [16]. It is a well-known marker of immune activation with high concentrations seen inmany inflammatory conditions, including infections, autoimmune disorders and cancer [16] [17] [18]. In humans, neopterin is known as a sensitive marker for infectious diseases associated with increased activity of the cellular immune system [19]. In one study it is found that serum neopterin levels are associated with COVID 19 disease activity and can be prognostic biomarker for its severity [20].

Nesfatin-1 is a neuropeptide produced in the hypothalamus and suppresses hunger feeding and increases insulin secretion from pancreatic beta islet cells. Therefore, nesfatin-1 has attracted attention as a new therapeutic agent in the treatment of obesity and diabetes mellitus, especially in thyroid diseases [21] [22] [23].

Since IL-17 and IL-23 are inflammatory cytokines, high concentrations of these markers arethought to be associated with the severity of HT and the progression of the disease, based on the autoimmune association between thyroid hormones, TPO, nesfatin-1 and neopterin. This study reveals whether these biomarkers can be used in patients with HT. Since HT remains a common disease with unknown pathogenesis, we aimed to determine the levels of these biomarkers in serum samples of patients with HT.

## Materials and methods

### Patients and control subjects

A total of 120 people, 90 patients who were diagnosed with HT by the Department of InternalMedicine, Harran University Faculty of Medicine, and 30 healthy people were participated in our study. Demographic data of the patients included in the study were recorded and detailed physical examinations were performed. The study was approved by the scientific ethics research coordinatorship of Harran University with the protocol number HRU/21.06.35 dated 15.03.2021.

The patients included in our study were divided into three groups. The first group consisted of 30newly diagnosed patients (20 men and 10 women) who had never been treated for HT. The mean age of these patients was 36.75±11.93 years. The second group consisted of 30 patients (8 males and 22 females) diagnosed with euthyroid HT and not treated with thyroid hormones before. The mean age of the patients was determined as 33.14±9.35 years. In the third group, there were 30 HT patients (9 men and 21 women) who were treated with levothyroxine, their mean age was 35.48±11.64 years. In addition, a healthy control group was formed with 30 individuals (12 men and 18 women) who did not have any autoimmune and chronic diseases. The mean age of these individuals was 39.00±8.46 years.

### Biochemistry analyzes

Thyroid stimulating hormone (TSH), free T3 (FT3; tri-iodothyronine), free T4 (FT4; thyroxine), thyroid peroxidase antibody (anti-Tpo), thyroglobulin antibody (anti-Tg), neopterin, nesfatin-1, IL-17 and IL-23 levels were measured in the serum samples of the participants.

Venous blood was drawn for laboratory tests after 8-12 hours of fasting for each participant. Measurements of the parameters from serum samples were determined by using Atellica (Elabscience Biotechnology, USA) instrument. Analyses were performed according to the manufacturers’ instructions for each ELISA kit. Optical density was read at 450 nm. The cutoff value of each assay was calculated according to the manufacturer’s instructions.

### Statistical analysis

Statistical analyses were carried out using the SPSS software 26.0 and Minitab 20.3. (IBM, Armonk, NY, USA). Descriptive statistics were provided as min, max, and meanstandard deviation. The Shapiro-Wilk test was preferred to check normality. Levene’s test was used to determine if the samples have equal variances. The Welch’s test for one-way ANOVA combined with the Games-Howell multiple comparison post-hoc test to perform pairwise comparisons across different groups was used. Cor relations among variables were determined using Pearson correlation. The values which are greater than 0.05 were considered not statistically significant.

## Results

The serum TSH, FT3, FT4, anti TPO, anti TG, neopterin, nesfatin-1, IL-17 and IL-23 levels of 120 individuals participating in the study are shown in Table 1. The age and gender distributions between the groups were similar (p=0.557) and (p=0.075) respectively.

**Table 1:** Clinical characteristics of HT patient and control groups^*^. ^*^ The number of subjects in each group is 30

Variables	Hypothyroidism			Euthyroid HT		
	Min	Max	x±SD	Min	Max	x±SD
Age (Year)	19.00	61.00	36.75±11.93	19.00	55.00	33.14±9.36
Neopterin (nmol/L)	18.43	24.65	21.49±1.76	7.53	12.74	10.49±1.36
Nesfatin-1 (nmol/L)	81.03	161.35	116.03±19.73	150.85	250.58	205.57±24.00
IL-17 (pg/mL)	10.45	291.39	187.53±46.60	27.34	84.56	51.22±12.96
IL-23 (pg/mL)	30.04	64.41	44.46±8.40	21.23	42.15	30.12±6.43
TSH (mU/L)	5.46	19.00	11.37±2.88	0.97	4.10	2.42±0.90
FT3 (pmol/L)	1.49	3.03	2.15±0.40	4.11	4.93	4.58±0.18
FT4 (nmol/L)	0.05	3.22	0.75±0.60	1.08	2.93	1.68±0.50
Anti-Tpo (U/mL)	600.00	1450.00	775.59±176.75	154.67	565.00	301.62±87.18
Anti-Tg (U/mL)	290.00	490.00	356.58±39.51	200.00	388.00	298.14±33.76
Variables	HT treated with levothyroxine			Healthy control group		
	Min	Max	x±SD	Min	Max	x±SD
Age (Year)	19.00	61.00	35.48±11.64	21.00	54.00	39.00±8.46
Neopterin (nmol/L)	7.51	12.03	9.37±1.21	2.90	6.94	4.74±1.16
Nesfatin-1 (nmol/L)	161.07	228.08	201.42±17.51	280.63	953.08	548.46±143.03
IL-17 (pg/mL)	62.54	89.65	75.98±7.15	23.25	39.79	30.73±4.63
IL-23 (pg/mL)	11.23	32.43	17.62±5.75	1.54	11.36	3.93±2.29
TSH (mU/L)	0.67	3.70	2.33±0.77	0.97	3.56	2.38±0.65
FT3 (pmol/L)	4.44	4.77	4.57±0.09	4.45	5.28	4.94±0.21
FT4 (nmol/L)	1.13	2.28	1.88±0.34	1.36	2.16	1.74±0.23
Anti-Tpo (U/mL)	86.34	397.05	182.96±85.71	21.00	54.00	29.23±7.58
Anti-Tg (U/mL)	70.00	116.00	88.14±8.64	1.20	7.50	2.42±1.65

### Cytokine quantification

Compared to the control group (30.73±4.63), IL-17 levels were found to be higher (p<0.001) in the hypothyroidism HT, euthyroid HT, and HT patients treated with levothyroxine groups (187.53±46.60, 51.22±12.95, and 75.98±7.15, respectively). Likewise, IL-23 levels were found to be higher in hypothyroidism HT (44.46±8.40 pg/mL) than the control group, euthyroid HT and HT patients treated with levothyroxine groups (3.93±2.29 pg/mL, 30.12±6.43 pg/mL, 17.62±5.75 pg/mL respectively) (p<0.001) (Table 1).

### Neopterin and Nesfatin-1 levels

Similaly, serum neopterin levels were found to be significantly high in hypothyroidism HT (p 0.001) but its levels in controls, euthyroid HT and HT patients treated with levothyroxine groups were as follows: (21.491.76 nmol/L 4.741.16 nmol/L, 10.491.36 nmol/L, 9.371.21 nmol/L respectively) Significantly lowest level of nesfatin-1 was found in HT whereas its levels in controls, euthyroid HT and HT patients treated with levothyroxine and hypothyroidism HT, groups were (548.46143.03 nmol/L, 205.5724.00 nmol/L, 201.4217.51 nmol/L 116.0319.73, nmol/L respectively) (p<0.001, Table 1).

Patients with high TSH levels were divided into three groups according to their symptoms and free T3 and free T4 levels. Newly diagnosed HT with hypothyroidism; TSH levels were above normal >10 mIU/L, and TPO and TG antibodies were positive.

Patients with subclinical hypothyroidism (euthyroid) HT and thyroid replacement therapy with levothyroxine, have normal TSH levels (<10 mIU/L), and TPO and TG antibodies are negative in these two groups of patients (Table 1).

The Welch’s ANOVA test revealed statistically significant differences between the four groups’means for neopterin, nesfatin, IL-17, and IL-23. (Neopterin - F (3, 64.16) = 650.9, p < 0.001; nesfatin- F (3, 62.55) = 195.55, p < 0.001, IL-17 - F (3, 58.38) = 369.96, p < 0.001, IL-23- F (3, 56.42) = 353.26, p < 0.001), indicating that the average hormone values measured in groups were not equal.

The Games-Howell Post-Hoc analysis results were presented in Table 2. The test yielded mean decreases in neopterin values from hypothyroidism, newly diagnosed HT to other groups, euthyroid HT, HT treated with levothyroxine, and healthy control group those were statistically significant (=0.00, =0.00, and =0.00), respectively. The test also provided mean decreases in terms of neopterin from euthyroid HT to other two groups, HT treated with levothyroxine, and healthy control group those were statistically significant (=0.00, and =0.00), respectively. An analysis of the means in HT treated with levothyroxine group and healthy control group were also significantly different as well (=0.00). The decrease in neopterin values between groups is also valid for other variables nesfatin-1, IL-17 and IL-23. Although the values of nesfatin-1 continued to decrease from euthyroid HT group to HT treated with levothyroxine group, they were not found statistically different (=0.88).

**Table 2:** The Games-Howell Post Hoc analysis results. ^*^ The mean difference is significant at the 0.05 level.

Variable	(I) group	(J) group	Mean Difference<br>(I-J)	Std. Error	p value
Neopterin	Healthy control<br>group	Euthyroid HT	11.00^*^	0.40	0.00
		HT treated with levothyroxine	12.12^*^	0.38	0.00
		Hypothyroidism, newly diagnosed HT	16.75^*^	0.38	0.00
	Euthyroid HT	HT treated with levothyroxine	1.11^*^	0.34	0.01
		Hypothyroidism, newly diagnosed HT	5.75^*^	0.33	0.00
	HT treated with	Hypothyroidism, newly diagnosed HT	4.63^*^	0.31	0.00
Nesfatin-1	Healthy control<br>group	Euthyroid HT	342.89^*^	25.67	0.00
		HT treated with levothyroxine	347.04^*^	25.49	0.00
		Hypothyroidism, newly diagnosed HT	432.44^*^	25.54	0.00
	Euthyroid HT	HT treated with levothyroxine	4.15	5.52	0.88
		Hypothyroidism, newly diagnosed HT	89.55^*^	5.73	0.00
	HT treated with	Hypothyroidism, newly diagnosed HT	85.40^*^	4.85	0.00
IL-17	Healthy control<br>group	Euthyroid HT	136.31^*^	8.58	0.00
		HT treated with levothyroxine	111.54^*^	8.34	0.00
		Hypothyroidism, newly diagnosed HT	156.79^*^	8.28	0.00
	Euthyroid HT	HT treated with levothyroxine	-24.77^*^	2.75	0.00
		Hypothyroidism, newly diagnosed HT	20.48^*^	2.55	0.00
	HT treated with	Hypothyroidism, newly diagnosed HT	45.25^*^	1.57	0.00
IL-23	Healthy control<br>group	Euthyroid HT	14.34^*^	1.91	0.00
		HT treated with levothyroxine	26.84^*^	1.83	0.00
		Hypothyroidism, newly diagnosed HT	40.53^*^	1.54	0.00
	Euthyroid HT	HT treated with levothyroxine	12.49^*^	1.60	0.00
		Hypothyroidism, newly diagnosed HT	26.19^*^	1.26	0.00
	HT treated with<br>levothyroxine	Hypothyroidism, newly diagnosed HT	13.69^*^	1.15	0.00

Our study showed that both serum levels of IL-23 and IL-17 were significantly increased in HT patients with newly diagnosed HT hypothyroid patients, euthyroid HT patients, and HT treated with levothyroxine patients respectively compared to the healthy control group.

### The correlations between parameters

Variables including neopterin, nesfatin-1, IL-17, IL-23, TSH, FT3, FT4, Anti-TPO, and Anti-TG were recruited into correlation analysis based on each group. Correlations of all variables were determined using Pearson correlation. No statistically significant correlation was found between any of these variables in the hypothyroidism group. The correlation coefficient between interleukin-17 and TSH was about r=-0.426 (p=0.021) indicating that there was a moderatenegative correlation in the euthyroid HT group. The correlation coefficient between neopterin andanti-TPO was about r=0.413 (p=0.026) indicating that there was a moderate positive correlation in the HT treated with levothyroxine group. In the same group, correlation coefficient between TSH and anti-TPO was also found significant r=0.519 (p=0.004). In the healthy control group, three statistically significant correlations were found. First correlation was between neopterin and nesfatin-1, r=0.427 (p=0.019) indicating that there was a moderate positive, as neopterin increases nesfatin-1 increases. Second correlation was between interleukin-23 and anti-TG, r=0.391 (p=0.033). Third correlation was between TSH and anti-TG, r=0.484 (p=0.007), Table 3.

**Table 3:** Pearson correlation coefficients of parameters based on four groups.

Neopterin	Nesfatin-1	IL-17	IL-23	TSH	FT3	FT4	Anti-TPO	Anti-TG	Groups
Neopterin	-0.236<br>(0.193)	-0.049<br>(0.790)	-0.258<br>(0.155)	-0.175<br>(0.337)	0.215<br>(0.237)	0.181<br>(0.322)	-0.105<br>(0.566)	-0.174<br>(0.341)	Healthy control
	-0.022<br>(0.911)	0.148<br>(0.445)	0.052<br>(0.788)	-0.283<br>(0.137)	0.032<br>(0.869)	-0.002<br>(0.991)	-0.035<br>(0.859)	-0.229<br>(0.232)	Euthyroid HT
	0.134<br>(0.488)	-0.060<br>(0.756)	-0.107<br>(0.582)	0.278<br>(0.145)	0.110<br>(0.571)	0.027<br>(0.888)	0.413^*^<br>(0.026)	-0.241<br>(0.207)	HT treated with<br>levothyroxine
	0.427^*^<br>(0.019)	0.236<br>(0.209)	0.063<br>(0.743)	-0.294<br>(0.114)	0.134<br>(0.479)	0.214<br>(0.257)	0.081<br>(0.671)	-0.282<br>(0.131)	Hypothyroidism
	Nesfatin-1	0.332<br>(0.063)	0.236<br>(0.193)	0.100<br>(0.585)	0.293<br>(0.103)	-0.245<br>(0.177)	0.179<br>(0.327)	-0.195<br>(0.285)	Healthy control
		0.311<br>(0.101)	0.031<br>(0.874)	-0.044<br>(0.822)	-0.229<br>(0.231)	-0.128<br>(0.507)	-0.028<br>(0.885)	-0.184<br>(0.340)	Euthyroid HT
		0.181<br>(0.348)	-0.180<br>(0.350)	-0.079<br>(0.684)	-0.287<br>(0.131)	-0.220<br>(0.253)	0.179<br>(0.354)	0.118<br>(0.541)	HT treated with<br>levothyroxine
		-0.022<br>(0.910)	-0.038<br>(0.842)	-0.291<br>(0.119)	0.092<br>(0.627)	0.203<br>(0.283)	0.146<br>(0.441)	-0.210<br>(0.266)	Hypothyroidism
		IL-17	0.288<br>(0.111)	0.257<br>(0.155)	0.087<br>(0.635)	0.212<br>(0.243)	-0.211<br>(0.246)	-0.107<br>(0.559)	Healthy control
			-0.044<br>(0.822)	-0.426^*^<br>(0.021)	-0.301<br>(0.112)	-0.346<br>(0.066)	0.036<br>(0.853)	-0.275<br>(0.149)	Euthyroid HT
			0.209<br>(0.276)	-0.085<br>(0.661)	-0.099<br>(0.608)	0.116<br>(0.550)	-0.153<br>(0.428)	-0.075<br>(0.700)	HT treated with<br>levothyroxine
			0.131<br>(0.492)	0.057<br>(0.766)	-0.284<br>(0.128)	0.253<br>(0.178)	0.193<br>(0.306)	0.040<br>(0.834)	Hypothyroidism
			IL -23	0.104<br>(0.570)	-0.024<br>(0.895)	-0.299<br>(0.097)	-0.183<br>(0.316)	-0.170<br>(0.353)	Healthy control
				-0.031<br>(0.871)	-0.035<br>(0.857)	-0.035<br>(0.856)	-0.177<br>(0.357)	0.136<br>(0.483)	Euthyroid HT
				-0.051<br>(0.794)	-0.043<br>(0.826)	-0.105<br>(0.588)	-0.361<br>(0.055)	0.015<br>(0.937)	HT treated with<br>levothyroxine
				0.192<br>(0.310)	0.007<br>(0.972)	-0.052<br>(0.784)	0.165<br>(0.383)	0.391^*^<br>(0.033)	Hypothyroidism
				TSH	0.065<br>(0.722)	0.264<br>(0.144)	-0.062<br>(0.736)	0.065<br>(0.725)	Healthy control
					-0.031<br>(0.872)	0.257<br>(0.179)	0.215<br>(0.263)	0.183<br>(0.341)	Euthyroid HT
					0.246<br>(0.198)	0.053<br>(0.783)	0.519^**^<br>(0.004)	0.069<br>(0.724)	HT treated with<br>levothyroxine
					0.154<br>(0.416)	-0.127<br>(0.504)	-0.064<br>(0.737)	0.484^**^<br>(0.007)	Hypothyroidism
					FT3	-0.093<br>(0.613)	0.153<br>(0.404)	-0.132<br>(0.471)	Healthy control
						0.302<br>(0.111)	-0.141<br>(0.465)	0.124<br>(0.521)	Euthyroid HT
						0.273<br>(0.151)	0.201<br>(0.296)	-0.070<br>(0.716)	HT treated with<br>levothyroxine
						-0.300<br>(0.108)	0.220<br>(0.242)	0.236<br>(0.209)	Hypothyroidism
						FT4	-0.241<br>(0.184)	-0.045<br>(0.805)	Healthy control
							0.361<br>(0.054)	0.345<br>(0.067)	Euthyroid HT
							-0.119<br>(0.538)	-0.095<br>(0.625)	HT treated with<br>levothyroxine
							0.028<br>(0.881)	0.088<br>(0.643)	Hypothyroidism
							Anti-TPO	0.154<br>(0.399)	Healthy control
								0.106<br>(0.584)	Euthyroid HT
								-0.070<br>(0.720)	HT treated with<br>levothyroxine
								0.098<br>(0.608)	Hypothyroidism

**Figure 1:**
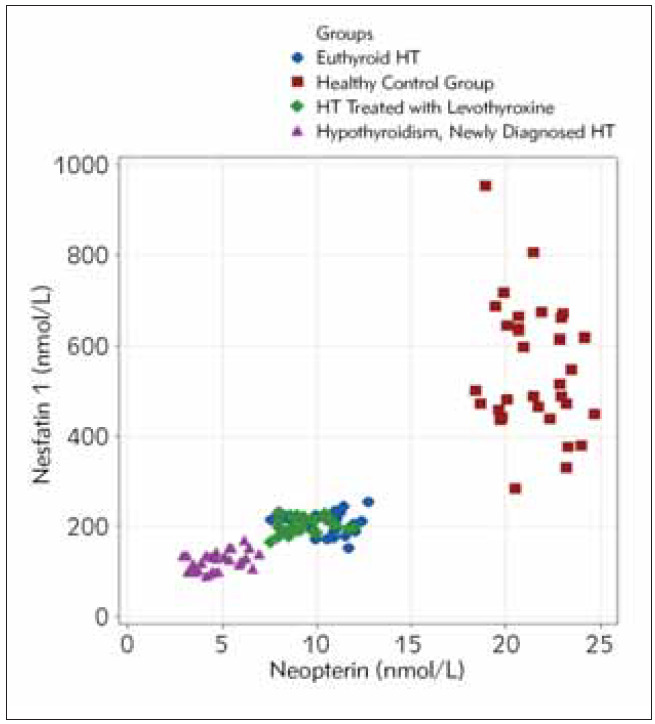
Scatterplot of Nesfatin-1 vs Neopterin for four groups.

The relationship between neopterin and nesfatin-1 in the healthy control group shown in Figure 1.

## Discussion

HT is a chronic autoimmune disease with a complex and heterogeneous course that primarily causes destruction and dysfunction of the thyroid gland [5]. Our study shows that both serum levels of IL-23 and IL-17 are significantly increased in HT patients with newly diagnosed HT hypothyroid patients, euthyroid HT patients, and HT treated with levothyroxine patients respectively compared to the healthy control group.

In HT, accumulation of lymphocytes in the thyroid gland eventually leads to thyroid fibrosis and gradual tissue destruction [5] [7] [24]. While the disease affects 2% of the general population, with atrend of increasing prevalence, women are five to ten times more likely to be affected [5] [24].

Recently, it has been suggested that Th3 and Th17 cells play an important role in the pathogenesisof chronic inflammatory diseases, including HT, chronic obstructive pulmonary disease (COPD), diabetes, and rheumatoid disease [10] [11] [25] [26] [27]. IL-23 is pro-inflammatory cytokine produced by macrophages and dendritic cells and responsible for many of the inflammatory autoimmune diseases [24] [28] [29]. Lymphoid cells secrete IL-17 cytokines upon IL-23 stimulation on them leading to enhanced expansion of T helper type 17 cells [30] [31]. Increased levels of IL-23 in HT lead to prolonged and high differentiation and proliferation of Th17 cells and increased inflammation [29]. These results suggest that IL-17 and IL-23 expression is increased under HT conditions and may play a role in the in its pathogenesis.

In our study, the increase in serum IL-23 levels in all three HT patient groups reflects that this IL-23has a role not only in the initiation of pathogenic processes but also in the maintenance of autoimmune inflammation. The correlation established between IL-17 and IL-23 for euthyroid patients confirms the positive association between these two cytokines and their involvement in disease onset. Our results showed significant differences in serum levels of IL-17 between healthy control and all HT patients. It decreased in patients with hypothyroid HT, which increased during treatment with levothyroxine, suggesting that IL-17 levels may be affected by hypothyroidism. Decreased IL-17 levels in hypothyroidism may be associated with depression of humoral and cell-mediated immunity in this functional state. These changes in serum levels of IL-17 were developed on the basis of general immune disorders found in the Th17 pathway in HT [25] [27] [30]. Taking into account other groups of HT patients, a significant increase in IL-17 and IL-23 levels was found in these patients (Table 2). IL-17 significantly intensifies the local inflammatory process by inducing T cell proliferation and B-lymphocyte differentiation [30] [31]. High IL-17 levels in hyperthyroidism affect the immune system and increase IL-23 levels. On the other hand, the use of levothyroxine in hyperthyroidism reduces IL-17 levels [32]. It has been shown that thyroxine suppression therapy used in hypothyroid patients affects the cellular immune reaction by increasing the levels of IL-17, soluble IL-23, and natural killer cells [14]. The results of above studies were found to be compatible with the results of ours.

It is well known that neopterin is proinflammatory marker synthesized by macrophages upon stimulation with gamma interferon-gamma and is indicative of cellular immune system activation. Levels of neopterin are elevated in conditions which has an immunological component such as autoimmune disease, viral and bacterial infections and malignancy [16] [33]. Neopterin levels in our study; It was found to be 21.491.76 nmol/L in HT patients, 10.491.36 nmol/L in euthyroid patients, 9.371.21 nmol/L in patients after treatment, and 4.741.16 nmol/L in the control group. Serum neopterin, was produced in greater amounts in HT and it seems to be a valid biological marker supporting the presence of HT. This situation has been attributed to the cellular immune system activation [17]. Recent study on Subacute thyroiditis although there is an inflammatory disorderand here is an increase in cytotoxic T cells in the thyroid gland but neopterin level was decreased [34]. In our study, serum neopterin levels in HT patients were significantly increased when compared to controls. However, our findings are in agreement with other studies in which increased serum neopterin level was observed in HT [18] [35].

Nesfatin-1 is satiety molecule produced in hypothalamus. It participates in the regulation of appetite and hunger [36]. It is involved in thyroid dysfunction body mass is reduced in hyperthyroidism whereas it is increased in hypothyroidism [23] [37]. It was found that dysfunction of thyroid hormones may effect food intake and weight [38]. In a study by Sawicka and Bossowski [22] showed that nesfatin-1 level was lower in subclinical hypothyroidism in HT and increased after l-thyroxine treatment. In our study, nesfatin-1 levels were variable in HT patients, the greatest value in clinically apparent ones and lower in subclinical hypothyroidism HT patients. In both groups the values were even lower. It has been suggested that thyroid hormones play significant role in the regulation the amount of nesfatin-1 secretion which in turn regulate the food intake and appetite. In our study, the levels of nesfatin-1 were found to be higher in hypothyroidism HT patients compared to the control group, while its levels were decreased in the HT treated with levothyroxine HT group. It was observed that nesfatin-1 decreased in patients with euthyroid HT. These results show that nesfatin-1 plays an important role in the body’s metabolic control mechanisms and can be used as a potential therapeutic agent in metabolic disorders, especially in HT patients having overweight [21] [22] [23]. We also think that the typical immune system disorder in such patients may have a role. A considerable number of people have positive TPO but with undiagnosed thyroid dysfunction [39]. Thus the measurement of the studied parameters may be helpful from diagnostic and prognostic point of view. It was reported that a monoclonal antibody directed against IL-23 have potential therapeutic effect on autoimmune disease by blocking IL-23 and indirectly affecting the production of IL-17 [27] [33]. Further studies are needed to determine antagonistic effect of such antibodies in treatment of HT.

## Conclusion

Our results suggest that the role of IL-23, IL-17 axis may play a role in the etiopathogenesis and development of HT in all stages of the disease as cause or effect. Moreover, our data show that the importance of IL-23 is more pronounced than IL 17 in the development and severity of HT. Increased neopterin production is also associated with HT and its levels correlate with the extent and the activity of the disease. Nesfatin-1 causes satiety. People with underactive thyroid gland would gain weight, and we always attribute this to slowing down of metabolism. Weight gain in this period may be related to increased appetite and may be due to low nesfatin-1 levels in these patients. This shows us that patients with Hashimoto’s thyroiditis are prone to obesity even if thyroid hormones do not decrease. Second, even if we correct the patient’s hormones, nesfatin-1 remains low compared to the health for these purposes. Further studies with better design are needed to be done on this subject.

## Dodatak

### Author contributions

All authors contributed to the writing of the initial draft. NB contributed to the discussion and edited the manuscript. NB and AÖ reviewed the manuscript. NB did the practical work in the lab. All authors have read and agreed to the published version of the article.

### Patient consent

Written consent has been obtained from each patient or subject after full explanation of the purpose and nature of all procedures used.

### Funding

This research was supported by the scientific research coordinatorship of Harran University, Turkey (Project no 21179).

### Ethical approval

The study was approved by the scientific ethics research coordinatorship of Harran University with the protocol number HRU/21.06.35 dated 15.03.2021.

### Conflict of interest statement

All the authors declare that they have no conflict of interest in this work.
